# Global trends and research hotspots in perioperative management of lung cancer: a bibliometric analysis from 2004 to 2024

**DOI:** 10.3389/fimmu.2024.1500686

**Published:** 2024-11-21

**Authors:** Qinling Jiang, Zhuheng Wei, Pingping Liu, Zonghuai Li, Huiqin Jiang, Yilin Cao, Bo Zhang, Yuanyuan Yan, Yulong He

**Affiliations:** ^1^ Department of Oncology, Nanxishan Hospital of the Guangxi Zhuang Autonomous Region, Guilin, China; ^2^ Scientific Research Center, Guilin Medical University, Guilin, China; ^3^ Department of Pharmacy, Sanya Central Hospital,The Third People’s Hospital of Hainan Province, Sanya, Hainan, China

**Keywords:** lung cancer, perioperative management, immunotherapy, targeted drugs, bibliometric

## Abstract

**Objective:**

This article aims to analyze the current status and research hotspots of literature related to perioperative management of patients with Lung Cancer and provide reference for future research directions.

**Methods:**

This study conducted a bibliometric analysis of research literature related to perioperative management of Lung Cancer published between 2004 and 2024, retrieved from the Web of Science database. R software and VOSviewer were used for analyzing keyword clusters and research themes, revealing trends and frontiers in this field.

**Results:**

A total of 4,942 studies on perioperative management of lung cancer were included. In recent years, research in this area has shown a global upward trend, with particular focus on surgical risk assessment, complication prevention, and postoperative management. Perioperative biomarkers before and after surgery have emerged as a central focus due to their impact on diagnosis and treatment. The application of novel therapies, such as targeted drugs and immunotherapy, in perioperative management is also becoming a significant research hotspot. Additionally, China has been a leading contributor to research output in this field, demonstrating strong performance in international collaborations.

**Conclusion:**

Perioperative management is a critical factor influencing the prognosis of Resectable lung cancer patients. Through a systematic analysis of the current status and research hotspots in perioperative management of lung cancer, this study provides valuable references for future clinical practice and research, particularly regarding the integration of novel therapies to optimize patient outcomes.

## 1 Introduction

Lung cancer is a malignancy that arises from cells in the lung tissue, typically originating from the cells lining the airways (1). Lung cancer is mainly divided into small cell lung cancer and non-small cell lung cancer (NSCLC), of which NSCLC accounts for 80%-85% (2, 3). Lung cancer is currently one of the most dangerous cancers globally, consistently ranking among the top three in incidence rates over the years (4). In 2022, there were approximately 19.97 million new cancer cases worldwide, with lung cancer accounting for 2.481 million cases, representing about 12.4% of all new cancer cases, once again making it the most common cancer (5). In China, lung cancer is both the most prevalent and the most deadly malignancy. According to epidemiological data from 2022, the incidence and mortality rates of lung cancer remain high, posing a serious threat to public health and presenting significant challenges to individuals and families (6).

Current treatments for lung cancer include surgery, radiotherapy, chemotherapy, targeted therapy, and immunotherapy. For early-stage NSCLC, surgery is usually the best treatment option (7). In addition, although surgery is the first choice for early-stage lung cancer, 30%-55% of patients still experience recurrence and death, so perioperative treatment is very important (8). In the past few decades, adjuvant platinum-based doublet chemotherapy after surgery has been the standard treatment for early-stage NSCLC patients. Although adjuvant chemotherapy has an advantage in disease-free survival (DFS) compared to surgery alone, the overall survival improvement is not significant, only increasing by 5% (9). Therefore, more effective treatments are urgently needed. In cases of advanced or inoperable lung cancer, chemotherapy are commonly used. With the rapid development of targeted and immunotherapy, these treatments have emerged as new approaches for lung cancer, particularly for patients with specific genetic mutations, such as EGFR or ALK mutations. Due to the high mortality rate of lung cancer (10), and the challenges posed by Locally advanced lung cancer, tumor biological heterogeneity, and patient individual differences, treatment remains highly challenging (11). In this context, perioperative management of lung cancer has become a critical component for successful treatment, highlighting its importance (12).

Perioperative management encompasses the entire treatment process, from preoperative assessment and preparation to intraoperative procedures and postoperative recovery (13). It is not just a time-specific concept but a comprehensive set of management measures aimed at maximizing surgical success and minimizing postoperative complications through multidisciplinary collaboration and integrated treatments, ultimately improving patient survival rates and quality of life (14–16).

Preoperative assessment involves several key aspects, such as determining whether the patient’s respiratory system can tolerate the surgical burden through tests like FEV1 (forced expiratory volume in one second) and DLCO (diffusing capacity of the lungs for carbon monoxide) (17). Preoperative interventions, including managing malnutrition, anemia, and chronic diseases, are also crucial, as improving these conditions can significantly enhance surgical safety and success rates (18). During the surgery, real-time monitoring of vital signs and oxygenation status is essential for ensuring surgical success. New surgical techniques, such as video-assisted thoracoscopic surgery (VATS), can greatly reduce the incidence of complications and accelerate postoperative recovery (19). Postoperative management is also vital for the long-term prognosis of lung cancer patients. Common postoperative complications include pulmonary infections, persistent air leaks, atelectasis, and arrhythmias (20). For some high-risk patients, postoperative treatments such as chemotherapy, radiotherapy, or targeted therapy are particularly important, as they can reduce the risk of tumor recurrence and improve survival rates. Additionally, regular follow-up and imaging surveillance are crucial for early detection of recurrences and timely intervention (21, 22). These processes can significantly speed up patient recovery and enhance prognosis.

While preoperative and postoperative care are vital components of lung cancer management, analyzing the academic research surrounding this topic provides a broader perspective on the field’s growth and progress. Bibliometrics is a widely used method for analyzing academic publications (23). With the advent of scientific databases such as Web of Science (WOS), obtaining research data has become more convenient, driving rapid developments in bibliometric research (24). As a comprehensive analytical method, bibliometrics combines quantitative and qualitative analysis, revealing various characteristics of publications, including identifying countries, journals, authors, and institutions that contribute significantly to the field, displaying frequently cited research and commonly used keywords, and uncovering collaboration relationships among countries, institutions, and authors within specific scientific domains (25). Furthermore, bibliometric methods provide an overview of the evolution and development frontiers of a research field for new researchers (26). However, there is currently a lack of bibliometric analysis on the perioperative literature in early-stage lung cancer. Although the number of relevant research papers has steadily increased, the knowledge system, research hotspots, and trend development in this field remain unclear. To fill this gap, this study employs R software, VOSviewer, and CiteSpace to conduct a systematic analysis of the literature on perioperative NSCLC from 2004 to 2024. We aim to explore the changes and development trends of research hotspots in this field and identify potential hotspots for future research. Looking ahead, a better understanding of the current state and potential of the perioperative management of lung cancer field is of great significance for its sustainable development.

## 2 Materials and methods

### 2.1 Data collection

The data used in this study were retrieved and downloaded from Web of Science database core data collection (WOSCC). (purchased version by Guangxi Medical University) on August 18, 2024. We used the following search formula:(((((TS=(“Preoperative Optimization” OR “Postoperative Care” OR “Perioperative Period” OR “Intraoperative Period” OR “Postoperative Period” OR “Neoadjuvant treatment” OR “Adjuvant treatment” OR “Neoadjuvant therapy” OR “Adjuvant therapy” OR Perioperative OR Neoadjuvant-adjuvant OR “Preoperative Period”)) AND TS=(“Pulmonary Neoplasm” OR “Lung Neoplasm*” OR “Lung Cancer*” OR “Cancer of Lung” OR “Pulmonary Cancer*” OR “Cancer of the Lung”))) AND DT=(Article OR Review)) AND LA=(English)) AND PY=(2004-2024).

After removing irrelevant literature, we reviewed 4,957 papers. After eliminating duplicates and retracted articles, a total of 4,942 papers were analyzed. The retrieved papers were saved in plain text format and exported as full records along with their cited references.

### 2.2 Data analysis

To analyze annual publications, Origin 2018 was used. Additionally, R software (version 3.6.3) along with the bibliometrix package (version 4.0) (27), VOSviewer (version 1.6.17) (28), and CiteSpace (version 6.1.4) (29)were employed for data visualization and to create scientific knowledge maps (30). To ensure data accuracy and reliability, data extraction and analysis management were performed by two different authors independently.

VOSviewer was used for visualizing co-authorship networks of countries/institutions, co-citation analysis of sources, and co-occurrence analysis of keywords. In the co-authorship network analysis, the following parameters were set: minimum number of publications for countries ≥25, and minimum number of publications for institutions ≥19. For the co-citation analysis of sources, the parameter set was: minimum number of citations for sources ≥120. Additionally, in the co-occurrence analysis of keywords, the parameters were set as follows: minimum number of occurrences for keywords ≥17. Journal impact factors (IFs) were retrieved from the 2023 Journal Citation Reports (JCR). The study introduced the Multiple Country Publication Ratio (MCP_Ratio) as an indicator to assess the degree of scientific collaboration among different countries. The calculation formula is as follows: MCP_Ratio = MCP/(SCP + MCP), where MCP represents publications involving multiple countries and SCP represents publications from a single country. Additionally, frequency (Freq) is used to indicate the proportion of articles from a specific country relative to the total number of articles, calculated using the formula: Freq = Articles/Total Articles. To further clarify the purposes and specific tasks of each analytical platform, we have summarized the applications of these tools in **Annex 1**.

## 3 Results

### 3.1 Overview of literature in the field of perioperative management of lung cancer

**Figure 1:**
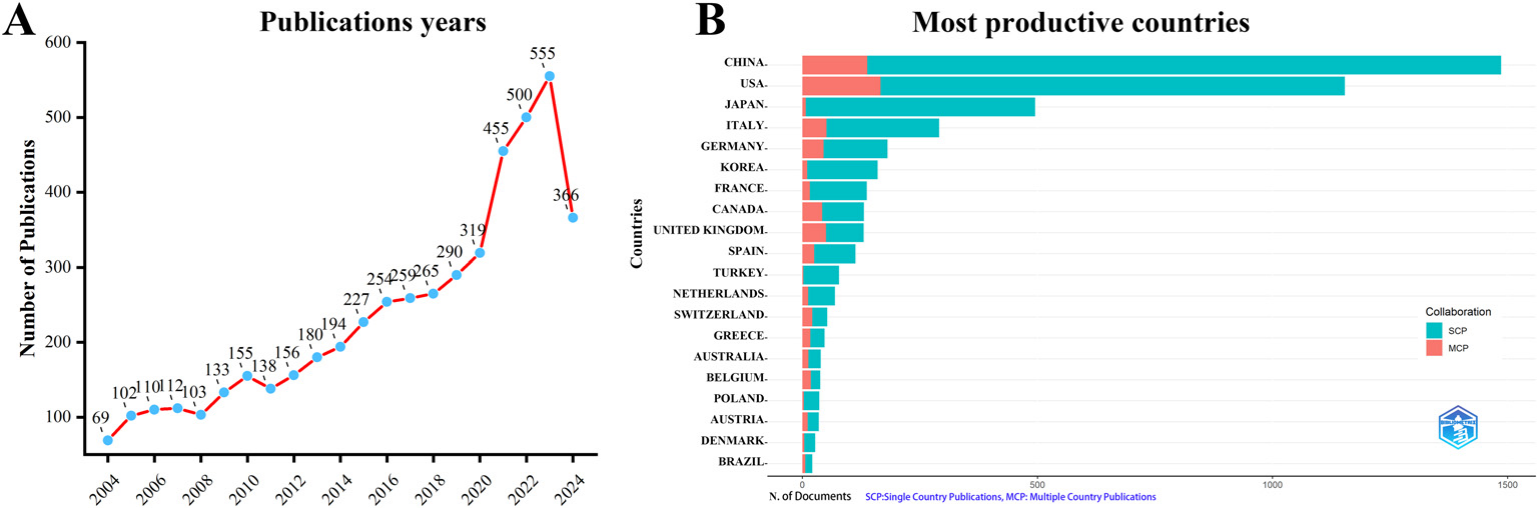
Annual publication trends in perioperative management of lung cancer research from 2004 to 2024 were analyzed.**(A)** Depicts the annual publication trends. **(B)** Illustrates the distribution of countries and the collaborative efforts among corresponding authors.

A total of 4,957 documents were collected from WoSCC. After removing duplicates and retracted articles, 4,942 documents remained. **Figure 1A** shows that the number of publications related to perioperative management of lung cancer has increased annually. There was a slow increase from 2004 to 2010, a moderate increase from 2010 to 2017, a rapid increase from 2017 to 2021, and an explosive growth from 2021 to 2023. In 2023, the number of published documents reached 555, and as of August 2024, 366 documents have been published in this field.

**Figure 2:**
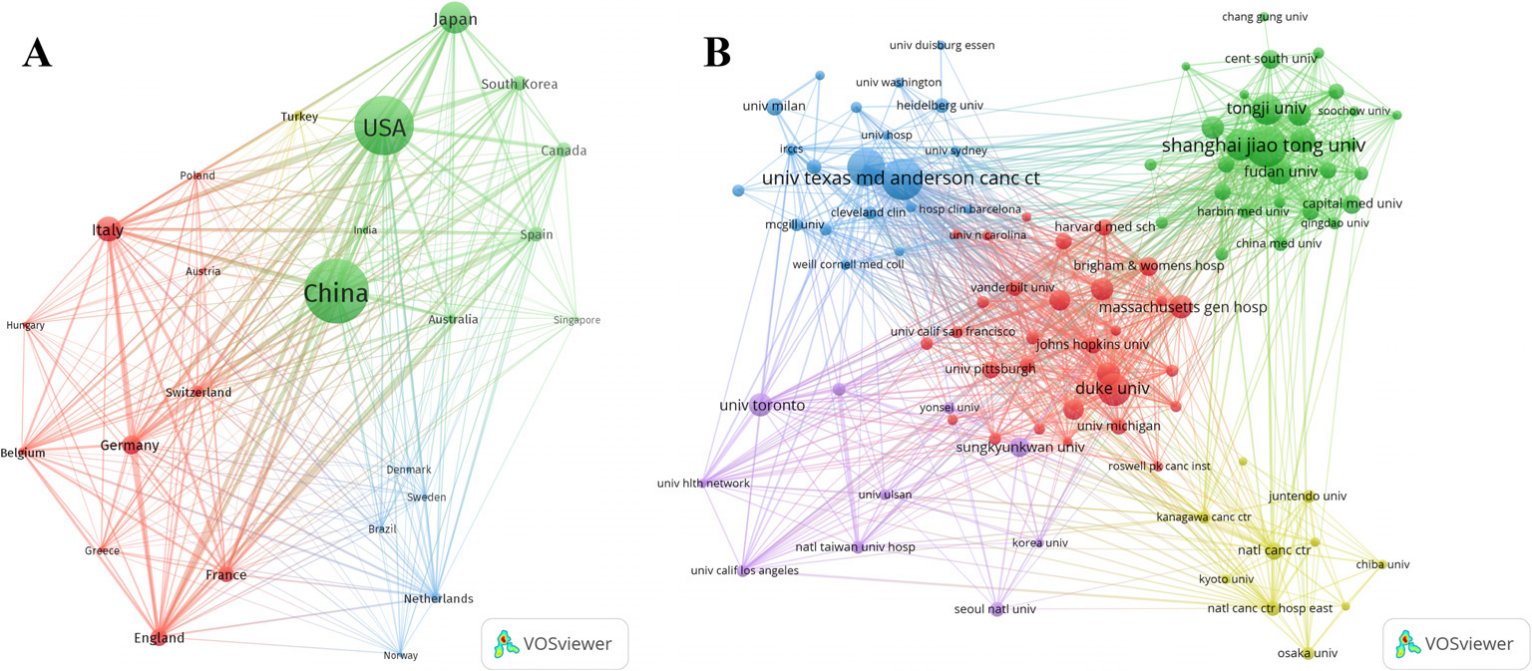
A map of countries involved in the field of perioperative management of lung cancer research from 2004 to 2024. **(A)** Map of cooperation between different countries. **(B)** Map of cooperation between different institutions.

According to the statistics based on the country of the corresponding authors, China (n = 1,481) has the highest productivity, followed by the USA (n = 1,148), Japan (n = 493), Italy (n = 290), and Germany (n = 181). Notably, among the top five countries by publication volume, China and the USA have multinational publication (MCP) ratios of 9.30% and 14.40%, respectively, which are significantly lower than those of Canada and the United Kingdom, with MCP ratios of 32.30% and 38.50% (**Figure 1B**, **Table 1**). Furthermore, **Figure 2A** indicates that China has the most extensive collaboration with other countries in the field of perioperative management of lung cancer research. Additionally, the collaboration map shows that The University of Texas System (n = 371) and Duke University (n = 148) are prominent centers of collaboration (**Figure 2B**; **Table 2**).

**Table 1:** Most relevant countries by corresponding authors.

Country	Articles	SCP	MCP	Freq	MCP_Ratio
China	1486	1348	138	0.300	0.093
USA	1154	988	166	0.233	0.144
Japan	495	488	7	0.100	0.014
Italy	291	240	51	0.059	0.175
Germany	181	136	45	0.037	0.249
Korea	160	150	10	0.032	0.063
France	137	121	16	0.028	0.117
Canada	131	89	42	0.026	0.321
United Kingdom	130	80	50	0.026	0.385
Spain	113	88	25	0.023	0.221
Turkey	78	76	2	0.016	0.026
Netherlands	69	57	12	0.014	0.174
Switzerland	53	32	21	0.011	0.396
Greece	47	30	17	0.009	0.362
Australia	39	26	13	0.008	0.333
Belgium	38	20	18	0.008	0.474
Poland	36	33	3	0.007	0.083
Austria	35	24	11	0.007	0.314
Denmark	27	23	4	0.005	0.148
Brazil	21	15	6	0.004	0.286

MCP, Multiple country publication; SCP, Single country publication.

**Table 2:** Most relevant affiliations in perioperative management of lung cancer.

Affiliation	Articles(n)
The University of Texas System	371
Harvard University	349
The University of Texas MD Anderson Cancer Center	309
Shanghai Jiao Tong University	234
Memorial Sloan Kettering Cancer Center	228
University of Toronto	199
Sichuan University	184
University of California	179
Sun Yat-sen University	171
Central South University	157
Chinese Academy of Medical Sciences (Peking Union Medical College)	150
Duke University	148
Fudan University	144
Tongji University	140
UNICANCER	139
Zhejiang University	136
Yale University	135
Tianjin Medical University	130
Sungkyunkwan University	129
National Taiwan University	123
Peking Union Medical College	118
Mayo Clinic	117
Ruprecht-Karls-University Heidelberg	116
Université Paris Cité	116
Harvard Medical School	114

### 3.2 Journals and co-cited journals

Using R software (version 3.6.3) with the Bibliometrix and ggplot2 packages, we analyzed the journals with the highest number of published articles and the most cited journals in this field. Additionally, VOSviewer (version 1.6.17) was utilized for co-citation analysis of journals. The results indicate that a total of 4,942 documents were published across 819 academic journals(**Annex 2**).

**Figure 3:**
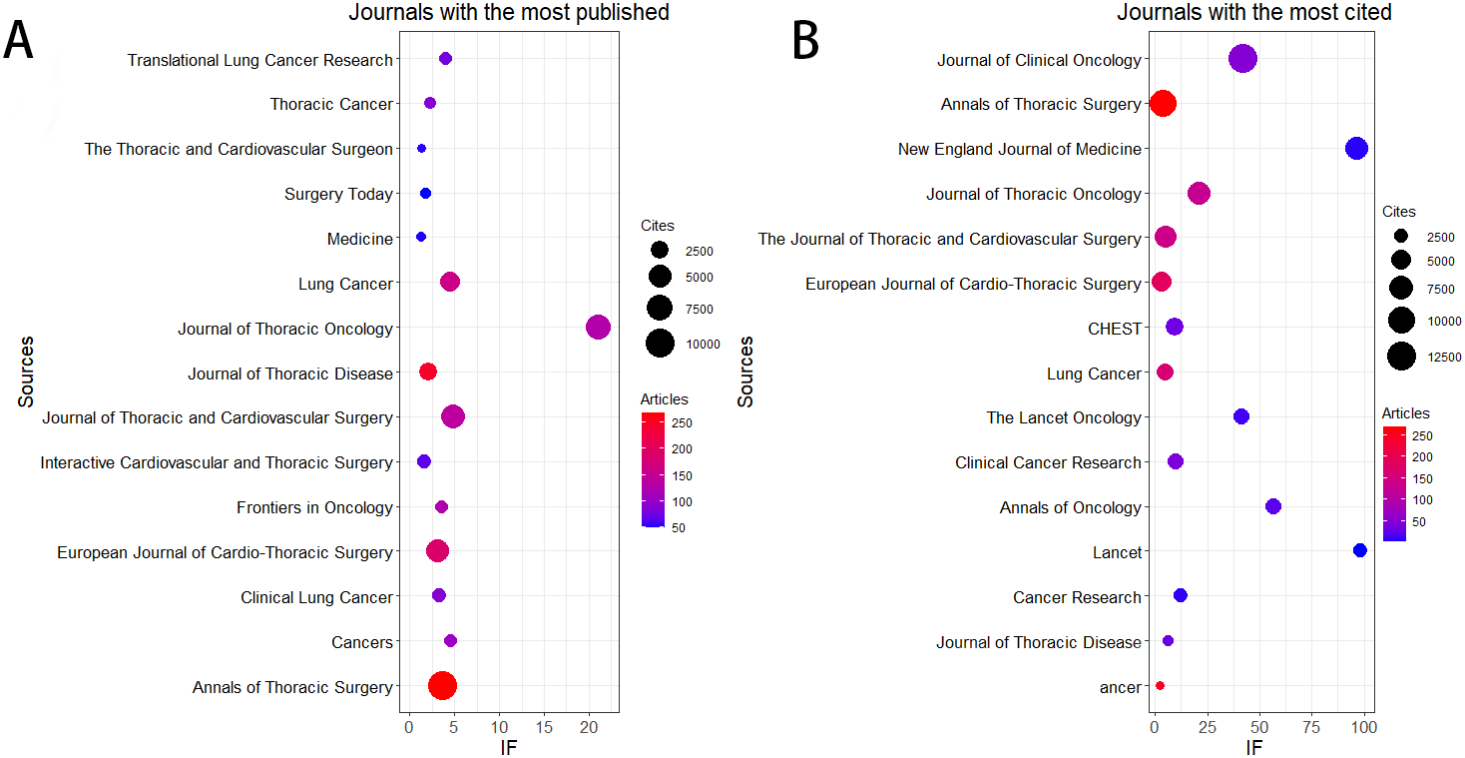
The journal with the highest volume of published articles and the journal with the most extensive citation count. **(A)** The journal with the highest quantity of published documents. **(B)** The journals with the most substantial citation counts.

**Table 3** and **Figure 3A** show that the journal with the highest number of published articles is the *Annals of Thoracic Surgery* (n = 268, IF = 3.6), followed by the *Journal of Thoracic Disease* (n = 247, IF = 2.1), the *European Journal of Cardio-Thoracic Surgery* (n = 186, IF = 3.1), *Lung Cancer* (n = 161, IF = 4.5), and the *Journal of Thoracic and Cardiovascular Surgery* (n = 139, IF = 4.9).

**Table 3:** Top 10 journals with the most published.

Sources	Documents	IF (2023)	Cites
Annals of Thoracic Surgery	268	3.6	10762
Journal of Thoracic Disease	247	2.1	2180
European Journal of Cardio-Thoracic Surgery	186	3.1	5381
Lung Cancer	161	4.5	3495
Journal of Thoracic and Cardiovascular Surgery	139	4.9	6243
Journal of Thoracic Oncology	126	21	6718
Frontiers in Oncology	123	3.5	804
Cancers	104	4.5	638
Clinical Lung Cancer	87	3.3	966
Thoracic Cancer	87	2.3	537

Additionally, **Table 4** and **Figure 3B** reveal that the most cited journal is the *Journal of Clinical Oncology* (n = 12,683, IF = 42.1), followed by the *Annals of Thoracic Surgery* (n = 10,762, IF = 3.6), the *New England Journal of Medicine* (n = 6,942, IF = 96.2), the *Journal of Thoracic Oncology* (n = 6,715, IF = 21), and the *Journal of Thoracic and Cardiovascular Surgery* (n = 6,243, IF = 4.9).

**Table 4:** Top 10 journals with the most cited.

Sources	Cites	IF (2023)	Documents
Journal of Clinical Oncology	12683	42.1	48
Annals of Thoracic Surgery	10762	3.6	268
New England Journal of Medicine	6942	96.2	6
Journal of Thoracic Oncology	6715	21	127
Journal of Thoracic and Cardiovascular Surgery	6243	4.9	139
European Journal of Cardio-Thoracic Surgery	5381	3.1	186
CHEST	3906	9.5	32
Lung Cancer	3495	4.5	161
Lancet Oncology	3377	41.6	12
Clinical Cancer Research	3281	10	42

**Figure 4:**
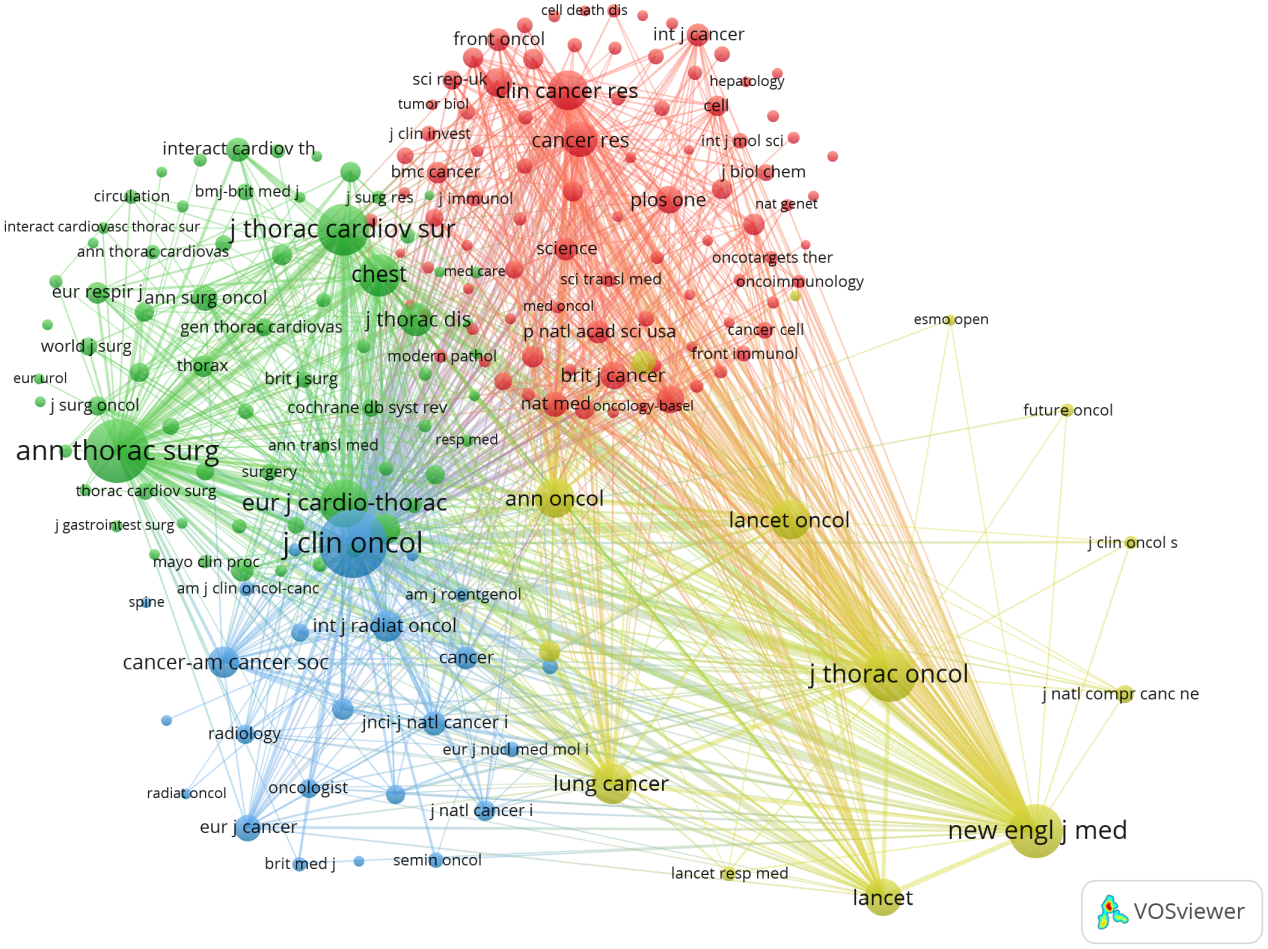
Co-cited journals related to perioperative management of lung cancer.

The co-citation analysis of journals shows that the *Journal of Clinical Oncology* and *Annals of Thoracic Surgery* are prominent centers of collaboration (**Figure 4**). Furthermore, we observed that the *Annals of Thoracic Surgery* and *Journal of Thoracic Disease* are among the top journals in both publication volume and citation count. These findings suggest that the *Annals of Thoracic Surgery*, *Journal of Clinical Oncology*, and *Journal of Thoracic Disease* are likely the most representative journals in this field. Additionally, these results highlight a relative scarcity of publications on perioperative achievements in top-tier journals, underscoring the need to enhance the depth and quality of research in this area.

### 3.3 Most cited references and reference burst

We used the Bibliometrix package in R software to identify the top 20 most cited references in perioperative management of lung cancer research (**Table 5**). These references each have over 400 citations and span 15 different journals, indicating that significant breakthroughs in this field are still emerging. Interestingly, no single journal dominates among the top 20 most cited references. The most cited references include “Integrated Genomic Characterization of Endometrial Carcinoma,” “A View on Drug Resistance in Cancer,” and “American Society of Clinical Oncology Treatment of Unresectable Non–Small-Cell Lung Cancer Guideline: Update 2003.” However, upon closer examination, we found that these articles are predominantly original research and review articles within the perioperative management of lung cancer research field.

**Table 5:** Top 20 cited references related to perioperative management of lung cancer.

Paper	DOI	Total Citations	TC per Year
GETZ G, 2013, NATURE	10.1038/nature12113	3652	304.33
VASAN N, 2019, NATURE	10.1038/s41586-019-1730-1	1412	235.33
PFISTER DG, 2004, J CLIN ONCOL	10.1200/JCO.2004.09.053	1173	55.86
WU YL, 2020, NEW ENGL J MED	10.1056/NEJMoa2027071	1001	200.20
HOWINGTON JA, 2013, CHEST	10.1378/chest.12-2359	964	80.33
HUNDAL R, 2014, CLIN EPIDEMIOL	10.2147/CLEP.S37357	731	66.45
CUZICK J, 2010, LANCET ONCOL	10.1016/S1470-2045(10)70257-6	685	45.67
CHAUDHURI AA, 2017, CANCER DISCOV	10.1158/2159-8290.CD-17-0716	635	79.38
YAMASHITA T, 2008, CANCER RES	10.1158/0008-5472.CAN-07-6013	608	35.76
BRUNELLI A, 2013, CHEST	10.1378/chest.12-2395	568	47.33
BURDETT S, 2014, LANCET	10.1016/S0140-6736(13)62159-5	564	51.27
WARTH A, 2012, J CLIN ONCOL	10.1200/JCO.2011.37.2185	549	42.23
VAN MEERBEECK JP, 2007, JNCI-J NATL CANCER I	10.1093/jnci/djk093	539	29.94
LARDINOIS D, 2006, EUR J CARDIO-THORAC	10.1016/j.ejcts.2006.08.008	519	27.32
PROVENCIO M, 2020, LANCET ONCOL	10.1016/S1470-2045(20)30453-8	480	96.00
KHORANA AA, 2005, CANCER-AM CANCER SOC	10.1002/cncr.21496	434	21.70
SCOTT WJ, 2007, CHEST	10.1378/chest.07-1378	433	24.06
CRINÒ L, 2010, ANN ONCOL	10.1093/annonc/mdq207	430	28.67
PILLAY B, 2016, CANCER TREAT REV	10.1016/j.ctrv.2015.11.007	415	46.11
ZHAO PF, 2019, J HEMATOL ONCOL	10.1186/s13045-019-0738-1	405	67.50

**Figure 5:**
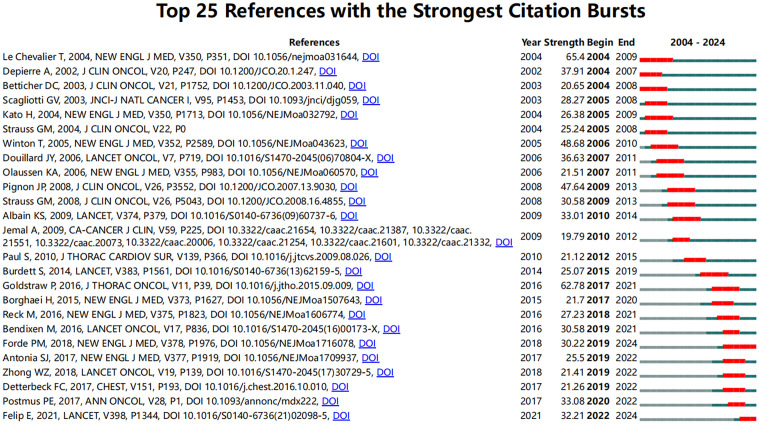
The top 25 most cited references on perioperative management of lung cancer.

To identify influential citation bursts in perioperative management of lung cancer research, we performed an analysis using CiteSpace (selection criteria: top 25; number of states: 2; minimum duration: 2). The results revealed 98 references showing significant citation bursts, with 25 of them illustrated in **Figure 5**. The top three references with the most active citation bursts are “Cisplatin-Based Adjuvant Chemotherapy in Patients with Completely Resected Non–Small-Cell Lung Cancer” (intensity: 65.4), “The IASLC Lung Cancer Staging Project: Proposals for Revision of the TNM Stage Groupings in the Forthcoming (Eighth) Edition of the TNM Classification for Lung Cancer” (intensity: 62.78), and “Vinorelbine Plus Cisplatin vs. Observation in Resected Non–Small-Cell Lung Cancer” (intensity: 48.68).

Notably, the three most recent citation bursts are “Adjuvant Atezolizumab After Adjuvant Chemotherapy in Resected Stage IB–IIIA Non-Small-Cell Lung Cancer (IMpower010): A Randomised, Multicentre, Open-Label, Phase 3 Trial,” “Neoadjuvant PD-1 Blockade in Resectable Lung Cancer,” and “Gefitinib Versus Vinorelbine Plus Cisplatin as Adjuvant Treatment for Stage II–IIIA (N1–N2) EGFR-Mutant NSCLC (ADJUVANT/CTONG1104): A Randomised, Open-Label, Phase 3 Study.” To gain deeper insights into the research frontiers and hotspots in perioperative management of lung cancer, we matched the DOIs of these 25 references with the titles in **Annex 3**.

In these referenced studies, randomized controlled trials (RCTs) constitute 27.3%, followed by phase studies (including Phase I, II, and III trials) at 22.7%, primarily focusing on the efficacy of various drugs during different stages of the perioperative period in lung cancer. These findings indicate that current research hotspots are centered on the combined use of neoadjuvant immunotherapy and chemotherapy, particularly in their application to NSCLC patients and their long-term prognostic outcomes.

### 3.4 Keyword clusters and evolution

Keyword clustering is an excellent way to understand the research hotspots and directions in a field. In this study, we extracted 6,467 keywords using VOSviewer. **Table 6** shows that the top 20 keywords each appeared more than 210 times. Among them, the most frequently occurring keyword is “survival” (n = 850), followed by “surgery” (n = 743), “cell lung-cancer” (n = 676), “chemotherapy” (n = 655), “resection” (n = 620), “lobectomy” (n = 366), “therapy” (n = 329), and “open-label” (n = 324).

**Table 6:** The top 20 keywords.

Rank	Keywords	Count
1	survival	850
2	surgery	743
3	lung-cancer	693
4	cell lung-cancer	678
5	chemotherapy	656
6	resection	622
7	lobectomy	366
8	therapy	329
9	open-label	324
10	carcinoma	299
11	outcomes	298
12	management	283
13	impact	267
14	mortality	260
15	cancer	256
16	trial	255
17	complications	250
18	radiotherapy	245
19	risk	233
20	adjuvant chemotherapy	229

**Figure 6:**
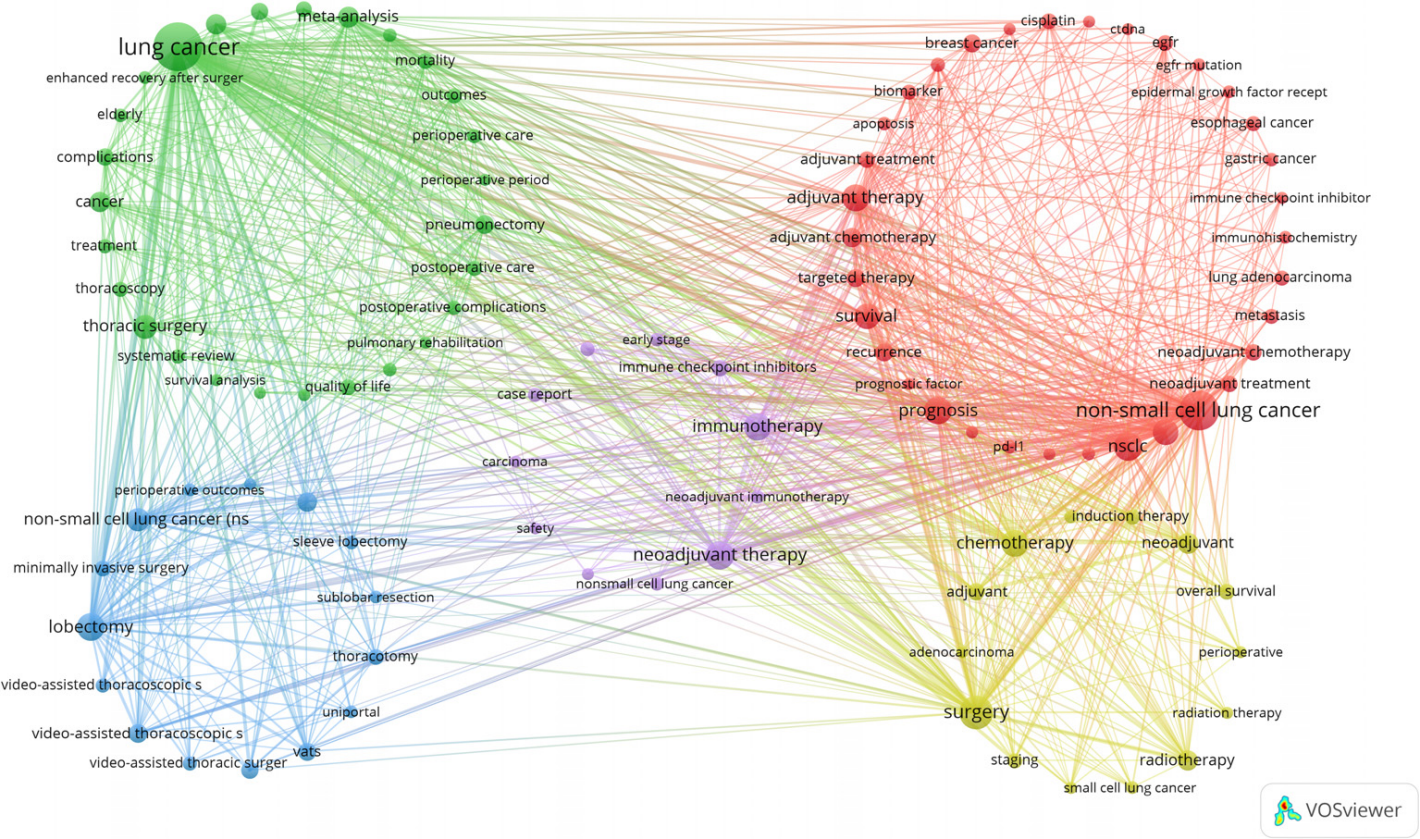
A co-occurrence map of keywords in the literature on perioperative management of lung cancer.

Next, we selected 101 keywords with a minimum occurrence of 17 times to create a keyword clustering map (**Figure 6**). In the map, we observed five distinct clusters represented by different colors. The tumor types and related genes cluster (red points) includes 34 keywords such as breast cancer, colorectal cancer, esophageal cancer, gastric cancer, and non-small-cell lung cancer. The lung cancer treatment and perioperative management cluster (green points) contains 27 keywords, including meta-analysis, morbidity, mortality, outcomes, perioperative care, and perioperative period. The lung cancer surgical techniques and minimally invasive treatments cluster (blue points) has 15 keywords, including robotic surgery, segmentectomy, sleeve lobectomy, sublobar resection, and thoracotomy. The lung cancer treatment strategies and prognosis cluster (yellow points) comprises 14 keywords such as therapeutic approaches, prognostic outcomes, oncological management, treatment modalities, and survival metrics. The lung cancer immunotherapy and clinical research cluster (purple points) includes 11 keywords like immune checkpoint inhibitors, immunotherapy, neoadjuvant immunotherapy, chemoradiotherapy, and case report (**Annex 4**).

**Figure 7:**
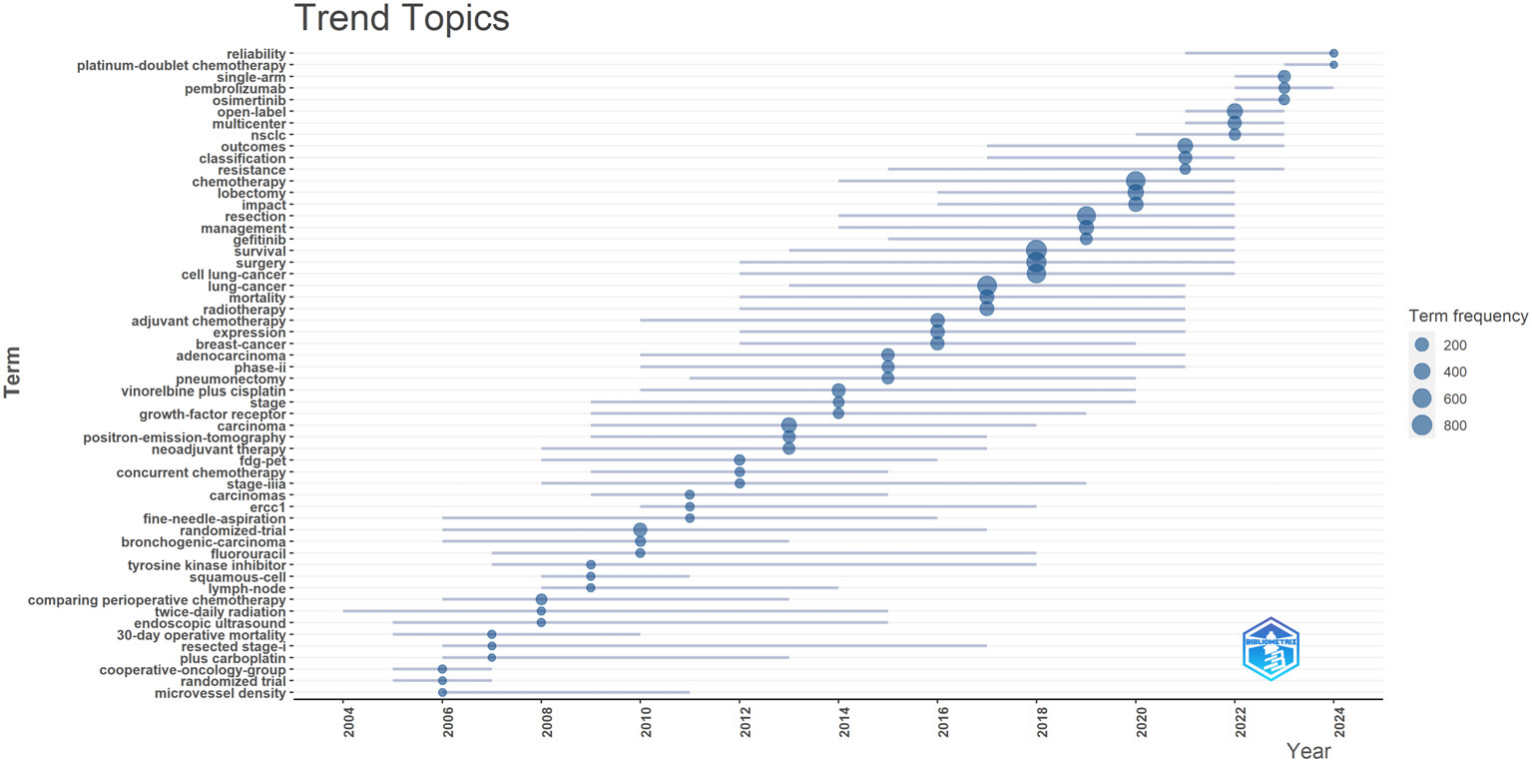
Trend topics in perioperative management of lung cancer research.

Additionally, we generated a trend topic map using the Bibliometrix package in R software (**Figure 7**). Trend topic maps are useful tools for identifying the chronological progression of research topics within a specific field, allowing us to examine the evolution of the field over time. By analyzing the trend topic map shown in **Figure 7**, we were able to identify the research focus and evolving trajectory of each stage in perioperative management of lung cancer research. Our findings indicate that the current research in this field mainly concentrates on pembrolizumab, open-label studies, and osimertinib.

Overall, our analysis reveals that recent lung cancer research has shifted focus toward novel targeted drugs and immunotherapy, with increased attention to postoperative management, survival rates, and new diagnostic technologies. In contrast, traditional randomized trials and some older keywords have seen a decline in focus.

## 4 Discussion

### 4.1 General information

In this study, we collected a comprehensive corpus of 4,942 documents covering the period from 2004 to 2024. The analysis indicates a continuous upward trend in literature on perioperative management of lung cancer, with a gradual increase from 2004 to 2010, a moderate rise from 2010 to 2017, a rapid surge from 2017 to 2021, and an explosive growth from 2021 to 2023. This phenomenon can be attributed to the following three reasons: (1) Breakthroughs in Immunotherapy: Recent advancements in immunotherapy, particularly the development of immune checkpoint inhibitors (ICIs), have significantly impacted perioperative management of lung cancer management. Clinical trials such as IMpower010 and CheckMate 816 have demonstrated the remarkable efficacy of immunotherapy in neoadjuvant and adjuvant settings. These studies not only improved patients’ disease-free survival rates but also spurred researchers to explore optimal use of immunotherapy, leading to an increase in related literature (31). (2) Diversification of Perioperative Treatments: The strategies for perioperative treatment have become increasingly complex, encompassing various combinations of immunotherapy, targeted therapy, chemotherapy, and radiotherapy. This diversification has not only broadened treatment options but also expanded the scope of research, contributing to the surge in literature (32). (3) Advancements in Precision Medicine: With the development of genomic sequencing technologies, researchers have identified more biomarkers associated with lung cancer, enabling more personalized treatments. The application of precision medicine has further fueled research enthusiasm and driven growth in literature in the related field. In the field of perioperative management of lung cancer research, China has emerged as a leading country, producing the highest number of academic publications. This trend reflects the significant impact of lung cancer on China, which has attracted considerable attention from Chinese researchers. A total of 4,942 documents are distributed across 818 journals, with notable contributions from prestigious publications such as *Annals of Thoracic Surgery*, *Journal of Thoracic Disease*, and *European Journal of Cardio-Thoracic Surgery*. In particular, *Annals of Thoracic Surgery* stands out for its substantial number of published articles and significant citation count. This prominent performance underscores *Annals of Thoracic Surgery* as a key publication in the field of perioperative management of lung cancer research, affirming its central role in disseminating research findings in this domain.

### 4.2 Hotspots and development trends

Through a comprehensive analysis of literature clustering, keyword frequency, keyword clustering, and thematic evolution, we have identified the potential hotspots in perioperative management of lung cancer research. The results indicate that the research frontiers and hotspots in this field are primarily focused on the following three aspects:

#### 4.2.1 An Examination of the efficacy of immunotherapy and adjuvant treatments in contemporary lung cancer management

Over the past 20 years, advancements in targeted therapy and immunotherapy have significantly improved the survival rates and prognosis for patients with advanced NSCLC. Recent studies highlight their potential not only in advanced stages but also in early-stage, surgically resectable NSCLC. Comprehensive perioperative strategies, particularly neoadjuvant and adjuvant therapies, are becoming key methods to reduce recurrence rates and enhance treatment outcomes.

Immunotherapy, especially immune checkpoint inhibitors (such as PD-1/PD-L1 inhibitors), has been shown to have important clinical value in the perioperative treatment of patients with early-stage NSCLC (33, 34). Its main mechanism is to enhance the patient’s immune system, identify and eliminate residual cancer cells, thereby reducing the risk of recurrence, including adjuvant immunotherapy and neoadjuvant immunotherapy (35).

Neoadjuvant immunotherapy (preoperative immunotherapy) is one of the focuses of current research. This model aims to reduce tumor size and improve resectability by using immunotherapy before surgery (36). Studies have shown that the combined use of immune checkpoint inhibitors and chemotherapy not only significantly improves the R0 resection rate (complete resection rate), but also significantly improves the Pathological Complete Response (pCR), indicating that tumor cells have been fully eliminated before surgery. For example, the CheckMate 816 study showed that nivolumab combined with chemotherapy effectively improved progression-free survival (EFS) by an average of about 10.8 months (31.6 months vs. 20.8 months, hazard ratio (HR) =0.63, P=0.005), while achieving complete pathological response. The rate reached 24%, which was significantly better than the 2.2% of chemotherapy alone (P<0.001) (37). In addition, neoadjuvant immunotherapy can also activate the patient’s immune system and help clear potential minimal residual disease after surgery, thereby reducing the risk of recurrence.

Postoperative adjuvant therapy is a strategy for patients who are not suitable for preoperative immunotherapy or have a small tumor burden. Postoperative adjuvant treatment (such as chemotherapy or immunotherapy) can effectively remove residual cancer cells and reduce the risk of recurrence (38). Especially for patients with low PD-L1 expression levels, postoperative immunotherapy can help enhance postoperative immune clearance and further prevent tumor recurrence. For example, the IMpower 010 study showed that atezolizumab as postoperative adjuvant therapy showed significant long-term benefit and prolonged DFS in patients with stage IB-IIIA NSCLC with high PD-L1 expression (39). In addition, pembrolizumab, as a PD-1 inhibitor, has also received good response in its application in metastatic NSCLC, supporting its potential in postoperative adjuvant therapy (40).

In order to maximize the therapeutic effect, when treating patients with NSCLC, the advantages of neoadjuvant and postoperative adjuvant therapy are usually combined, and immunotherapy and chemotherapy are used before and after surgery to minimize the possibility of recurrence, e.g. A clinical trial evaluating tolipalumab in combination with platinum-based chemotherapy in patients with resectable stage III NSCLC showed that compared with chemotherapy alone, tolipalumab significantly prolonged Event-free survival (EFS) and major pathological response rate(MPR), and the combined treatment regimen is controllable in terms of safety, providing a new adjuvant treatment option for this patient group (41, 42). This comprehensive treatment strategy is especially suitable for patients at high risk of recurrence and is one of the important directions for future lung cancer treatment.

Targeted therapy also provides notable benefits in the perioperative treatment of lung cancer, especially in NSCLC patients. Targeted therapy typically includes neoadjuvant therapy before surgery and adjuvant therapy after surgery, targeting specific genetic mutations (such as EGFR mutations) and demonstrating multifaceted advantages (43). The ADAURA study demonstrated that postoperative use of osimertinib significantly prolonged DFS and overall survival, with DFS benefits translating into statistically significant OS improvements, suggesting that adjuvant osimertinib can substantially reduce the risk of recurrence and improve long-term survival rates (44). However, the neoadjuvant use of osimertinib alone has shown suboptimal results (MPR 15%, pCR 0), and the benefits of combining osimertinib with chemotherapy in the neoadjuvant setting are still pending confirmation from the neoADAURA trial (45). As for the efficacy of neoadjuvant immunotherapy combined with chemotherapy in EGFR-mutant patients, there is currently no prospective evidence to suggest that perioperative immunotherapy benefits patients with resectable, driver mutation-positive lung cancer (46). While subgroup analyses from studies like Keynote671 indicated that EGFR-mutant patients had a better HR compared to wild-type patients (HR 0.09 vs. 0.48), smaller single-arm studies such as the NEOTIDE trial, which reported an MPR of 45% and pCR of 5%, are insufficient to establish the role of immunotherapy in EGFR-mutant patients during the perioperative period (47). Thus, while postoperative targeted therapy significantly improves survival, neoadjuvant targeted therapy has not shown the same benefits. For EGFR-positive patients, the optimal neoadjuvant treatment strategy, whether combining targeted therapy with chemotherapy or immunotherapy with chemotherapy, remains unclear and requires further research.

Similarly, targeted therapy has also demonstrated its efficacy in ALK-positive patients. The ALINA study further validated this by showing that, in patients with stage IB to IIIA ALK-positive NSCLC, adjuvant treatment with alectinib after complete resection reduced the risk of disease recurrence by 76% compared to chemotherapy (DFS HR=0.24, 95% CI 0.13–0.43, P<0.0001). Notably, the median DFS in the alectinib group has not yet been reached, while in the chemotherapy group, it was 41.3 months. Although overall survival data are still immature, the significant improvement in DFS highlights that alectinib is a safe and effective postoperative adjuvant therapy option for these patients (38).

However, it is important to note several limitations regarding the use of these therapies in perioperative management of lung cancer: (1) Immunotherapy may be ineffective in some patients due to Primary resistance, which can affect treatment efficacy (48, 49). (2) Due to serious adverse reactions of drugs, patients miss the opportunity for surgical treatment. (3) The impact of perioperative treatments is complex, and many mechanisms still require further investigation by researchers.

#### 4.2.2 Novel biomarkers in perioperative assessment

Through the analysis of existing literature, circulating tumor DNA (ctDNA) and circulating tumor cells (CTC) have emerged as research hotspots in perioperative biomarkers for lung cancer in recent years. These biomarkers play a crucial role in the early diagnosis, treatment planning, monitoring of therapeutic response, and postoperative prognosis assessment of lung cancer.

The ctDNA, which consists of tumor DNA fragments present in the bloodstream, has shown great potential in tracking minimal residual disease and predicting tumor recurrence (43). The dynamic changes in ctDNA levels can reflect tumor burden, making monitoring during treatment highly significant. Studies have indicated that if ctDNA is not cleared in NSCLC patients following perioperative treatment, their risk of recurrence significantly increases, suggesting the need for adjustment in the treatment plan (50, 51). As a non-invasive biomarker, ctDNA is particularly critical in the early detection of postoperative recurrence due to its rapid and sensitive characteristics (52, 53).

Minimal Residual Disease (MRD) refers to the small number of cancer cells that persist in a patient’s body after treatment, which are often undetectable by standard diagnostic methods but can lead to disease relapse (54). The detection of MRD is crucial for evaluating the effectiveness of cancer therapy and guiding subsequent treatment decisions. Techniques such as flow cytometry, polymerase chain reaction, and next-generation sequencing are employed to identify these residual cells and assess their presence with high sensitivity (55). Monitoring MRD helps in predicting the risk of disease recurrence and tailoring treatment plans to prevent relapse, thus improving patient outcomes and advancing personalized medicine strategies (56).

Furthermore, the evaluation of postoperative inflammatory response plays an important role in cancer prognosis (57–59). Studies have shown that postoperative inflammatory status is closely related to the risk of cancer recurrence (60). Inflammatory markers such as C-reactive protein (CRP) and white blood cell count provide valuable information in postoperative monitoring (61). Moreover, some studies have found that elevated levels of postoperative inflammatory markers (such as IL-6) are associated with an increased risk of recurrence in NSCLC patients (62). Therefore, assessing postoperative inflammatory response not only helps in predicting the risk of recurrence but also may provide a basis for developing individualized treatment plans.

However, there are certain limitations: (1) Although ctDNA shows potential in tumor monitoring, the existing detection methods and standardized procedures for ctDNA still exhibit significant variability. Different detection methods and analytical platforms may lead to variations in results (63). While CTC detection is important for understanding cancer metastasis, its clinical application is limited by the sensitivity and specificity of current detection techniques (64).

#### 4.2.3 Perioperative complications and prognosis in lung cancer

Lung cancer surgery-related perioperative complications and prognosis constitute a critical area of research within the field, as they profoundly influence both short-term recovery and long-term survival outcomes for patients. Perioperative complications, particularly air leaks, pleural effusions, pulmonary infections (50), and pulmonary thromboembolism (65), not only prolong hospitalization but may also lead to significant long-term health problems and a marked decline in quality of life.

In terms of prognosis, the occurrence of perioperative complications is closely related to patients’ long-term survival and disease-free survival rates. Studies have shown that postoperative pulmonary complications can directly affect recovery and may indirectly influence tumor recurrence and overall prognosis through the induction of systemic inflammatory responses (66).

There are various strategies to prevent these complications, with patient nutrition being of paramount importance. Low protein levels and malnutrition have been found to be closely associated with a higher incidence of postoperative complications, particularly among elderly patients. Malnutrition can lead to poor tissue healing and weakened immune function, thereby increasing the risk of infections and other complications. For instance, research indicates that patients with a lower Prognostic Nutritional Index (PNI) are more prone to persistent air leaks, pneumonia, and other infectious complications (66–68).

The complexity and duration of surgery are also significant factors influencing complications. Longer surgical durations are typically associated with higher surgical invasiveness (69), such as thoracotomy, which causes greater damage to the chest wall and increases the risk of postoperative pulmonary complications (70).

Traditionally, patients were required to fast for at least six hours before surgery. However, recent studies have shown that moderate preoperative carbohydrate intake can significantly reduce postoperative endocrine responses. As a result, current guidelines allow the intake of clear liquids up to two hours before surgery (71). Additionally, regional anesthesia is an effective technique that can reduce endocrine metabolic responses and provide postoperative analgesia (72). Compared to general anesthesia, regional anesthesia significantly lowers the incidence of postoperative complications (73). Smoking markedly increases the risk of postoperative pulmonary complications (74); therefore, preoperative smoking cessation is an essential measure to improve lung health (75, 76). Overall, implementing these perioperative interventions can significantly enhance the outcomes of lung cancer surgery.

However, there are some limitations to these findings: (1) The occurrence of complications and prognosis may be influenced by various factors, such as underlying diseases and lifestyle habits, which many studies may not have adequately controlled for. (2) Many studies may focus only on short-term outcomes, neglecting long-term prognosis. For example, the long-term effects of perioperative complications may require longer follow-up to evaluate.

### 4.3 Future directions and limitations

#### 4.3.1 Future directions

Pathological complete response of existing neoadjuvant immunotherapy combination chemotherapy regimens in NSCLC is approximately 20%, but there are significant differences across different molecular subtypes. Patients with KRAS and EGFR mutations respond particularly differently to neoadjuvant therapy. Future research should concentrate on optimizing treatment options based on the molecular characteristics of tumors. For instance, for patients with KRAS mutations, the emergence of KRAS G12C inhibitors provides new hope for enhancing perioperative outcomes. By combining KRAS inhibitors with immunotherapy or chemotherapy, it may be possible to significantly improve the pCR rate and reduce the risk of postoperative recurrence (77). For patients with EGFR mutations, although the results of current neoadjuvant treatments are suboptimal (for example, the pCR rate of osimertinib treatment is 0% and the MPR rate is only 15%), combining with new targeted drugs or signaling pathway modulators may yield better results in the future (45). Strengthening molecular testing and clarifying different gene mutation types and their treatment responses will lay the foundation for further optimization of perioperative treatment strategies.

KRAS mutation is one of the most prevalent driver mutations in NSCLC, especially the KRAS G12C mutation, which is continuously activated via the RAS-MAPK signaling pathway and promotes the proliferation and survival of tumor cells. Persistent activation of this pathway not only increases tumor aggressiveness but also leads to resistance to traditional therapies such as chemotherapy (78). In recent years, the development of KRAS inhibitors (such as KRAS G12C inhibitors) has demonstrated initial success in treating patients with such mutations. Future research should focus on exploring the combined application of KRAS inhibitors and other therapies (such as immune checkpoint inhibitors and chemotherapy), particularly in the perioperative stage, using multiple strategies to inhibit the KRAS pathway and its related drug resistance mechanisms, thereby enhancing treatment efficacy before and after surgery (79). Additionally, further investigation into the cross-regulatory mechanisms of the RAS-MAPK pathway will help to target therapeutic interventions more effectively for patients with KRAS mutations (80).

EGFR mutations also play a crucial role in NSCLC, particularly in non-smoking patients. These mutations regulate the growth, proliferation, and survival of tumor cells through abnormal activation of the EGFR signaling pathway, which includes critical downstream pathways such as PI3K/AKT and JAK/STAT (81). Current EGFR-targeted therapies have achieved favorable clinical outcomes in postoperative adjuvant therapy, but their efficacy in neoadjuvant therapy is limited. This limitation may be linked to the complexity of the EGFR signaling pathway, variations in the tumor microenvironment, and resistance to immunotherapy (82). Therefore, future research should concentrate on developing new EGFR-targeting drugs or combination treatment regimens to improve perioperative treatment effects. Specifically, combining the inhibition of key nodes downstream of EGFR, such as the PI3K/AKT pathway, may yield better results during the neoadjuvant treatment phase (83).

For patients with KRAS and EGFR mutations, optimizing treatment models will be a key focus for future research. Comparative studies between neoadjuvant + surgery + adjuvant (sandwich model) and neoadjuvant treatment alone will help to determine the best perioperative treatment pathway. For patients with KRAS mutations, a strategy that combines KRAS inhibitors with immunotherapy or chemotherapy is anticipated to increase the surgical resection rate and reduce the risk of recurrence (84). For patients with EGFR mutations, in-depth research into the best targeted treatment options before and after surgery, especially through enhanced molecular testing and signaling pathway analysis, can help formulate more individualized treatment plans to further improve the pCR rate and overall survival rates (85).

#### 4.3.2 Limitations

This study provides researchers with deeper insights into the perioperative field of lung cancer and explores new research directions. However, there are several limitations to this study.

First, despite our efforts to cover common terms related to perioperative management and lung cancer, different researchers may use expressions not included in our search, potentially leading to missed literature. In our literature search, we carefully selected terms associated with perioperative management and lung cancer. For perioperative management, we utilized terms such as “Preoperative Optimization,” “Postoperative Care,” and “Perioperative Period” to encompass the main phases: preoperative, intraoperative, and postoperative. Additionally, we included widely used terms like “Neoadjuvant Treatment” and “Adjuvant Treatment” to ensure we captured standard expressions for perioperative interventions. To further expand our search, we incorporated broader terms such as “Neoadjuvant-Adjuvant” and “Perioperative”. Regarding lung cancer-related terms, we employed common keywords like “Lung Cancer*” and “Pulmonary Neoplasm*” and included synonyms (e.g., “Cancer of Lung”) to ensure comprehensiveness. This diverse search strategy aims to reduce potential biases in terminology usage due to regional or disciplinary differences, ensuring that relevant literature is not overlooked. Second, we relied solely on the WoSCC database as our data source, which may have led to the exclusion of some publications from our analysis. Nevertheless, the WoS database is widely recognized as a high-quality digital literature repository and is considered one of the best options for bibliometric analysis (86–88). Therefore, our choice of data source is reliable. Additionally, our analysis was limited to English publications, which may introduce source bias. Lastly, while China faces a significant burden from lung cancer and has extensive research on this topic, we did not analyze Chinese literature, which may result in minor biases in our data analysis (89).

## 5 Conclusion

Our study clearly outlines the key research hotspots and frontiers in the field of perioperative management of lung cancer research. The following is a summary of the key points and research trends in this area:

a. Perioperative management of lung cancer research has garnered global attention, with China, the United States, Japan, and Italy being the most active countries. Extensive international collaboration has been observed among these nations.

b. *Annals of Thoracic Surgery* and *Journal of Thoracic Disease* are the most prominent journals for publishing perioperative management of lung cancer-related literature. Notably, *Annals of Thoracic Surgery* has the highest citation rate, indicating its significant influence in the field of perioperative management of lung cancer research.

c. In the perioperative management of lung cancer, the combination of adjuvant chemotherapy, targeted therapy, and immunotherapy represents a current hotspot and trend in surgical treatment.

d. The evaluation of biomarkers such as ctDNA and CTCs, as well as postoperative inflammation, is a rapidly emerging trend for facilitating personalized treatment plans in perioperative management of lung cancer management.

e. Research on complications and prognosis during the perioperative period, including air leaks, pleural effusion, pulmonary infections, and pulmonary embolism, represents a key focus and trend in the study of perioperative complications.

f. Future research should focus on the following key areas: enhancing the effectiveness of pCR and personalized treatment, investigating the impact of KRAS mutations and their associated signaling pathways on tumor biology, exploring the mechanisms of EGFR mutations and their signaling pathways, and optimizing perioperative treatment models.

Our research provides a comprehensive insight into the research trends and hotspots in the field of perioperative management of lung cancer. These findings not only enhance researchers’ understanding of the field but also guide future exploration and innovation. By delineating current research patterns and potential focus areas, our study offers a valuable theoretical foundation and practical guidelines, supporting scholars in conducting more systematic and pioneering research in this critical area.

## Data Availability

Publicly available datasets were analyzed in this study. This data can be found here: Available through the following URL: https://www.webofscience.com.
