# Supplementary material for: Global trends and research hotspots in perioperative management of lung cancer: a bibliometric analysis from 2004 to 2024
Source: Front Immunol. 2024 Nov 21;15:1500686. doi: 10.3389/fimmu.2024.1500686 (PMC11617563; doi:10.3389/fimmu.2024.1500686)
Supplement: Supplementary file 4 [file Table4.docx]

Cluster 1

adjuvant chemotherapy

adjuvant therapy

adjuvant treatment

apoptosis

biomarker

biomarkers

breast cancer

circulating tumor dna

cisplatin

colorectal cancer

ctdna

egfr

egfr mutation

epidermal growth factor receptor

esophageal cancer

gastric cancer

immune checkpoint inhibitor

immunohistochemistry

lung adenocarcinoma

metastasis

neoadjuvant chemotherapy

neoadjuvant treatment

non-small cell lung cancer

non-small-cell lung cancer

nsclc

osimertinib

pancreatic cancer

pd-l1

positron emission tomography

prognosis

prognostic factor

recurrence

survival

targeted therapy

Cluster 2

cancer

complications

elderly

enhanced recovery after surgery

lung cancer

lung cancer surgery

lung neoplasms

lung resection

meta-analysis

morbidity

mortality

outcomes

perioperative care

perioperative period

pneumonectomy

postoperative care

postoperative complications

pulmonary rehabilitation

pulmonary resection

quality of life

risk factors

surgical resection

survival analysis

systematic review

thoracic surgery

thoracoscopy

treatment

Cluster 3

lobectomy

minimally invasive surgery

non-small cell lung cancer (nsclc)

perioperative outcomes

robotic surgery

segmentectomy

sleeve lobectomy

sublobar resection

thoracotomy

uniportal

vats

video-assisted thoracic surgery

video-assisted thoracic surgery (vats)

video-assisted thoracoscopic surgery

video-assisted thoracoscopic surgery (vats)

Cluster 4

adenocarcinoma

adjuvant

chemotherapy

disease-free survival

induction therapy

neoadjuvant

overall survival

perioperative

radiation therapy

radiotherapy

small cell lung cancer

stage iii

staging

surgery

Cluster 5

carcinoma

case report

chemoradiotherapy

early stage

immune checkpoint inhibitors

immunotherapy

neoadjuvant immunotherapy

neoadjuvant therapy

nonsmall cell lung cancer

prognostic factors

safety
